# Road-Aided Ground Slowly Moving Target 2D Motion Estimation for Single-Channel Synthetic Aperture Radar

**DOI:** 10.3390/s16030383

**Published:** 2016-03-16

**Authors:** Zhirui Wang, Jia Xu, Zuzhen Huang, Xudong Zhang, Xiang-Gen Xia, Teng Long, Qian Bao

**Affiliations:** 1 Department of Electronic Engineering, Tsinghua University, Beijing 100084, China; zhirui1990@126.com; 2 School of Information and Electronics, Beijing Institute of Technology, Beijing 100081, China; hzzhit@126.com (Z.H.); longteng@bit.edu.cn (T.L.); 3 Department of Electrical and Computer Engineering, University of Delaware, Newark, DE 19716, USA; xianggen@gmail.com; 4 Insititute of Electronics, Chinese Academy of Sciences, Beijing 100190, China; baoqiancherry@163.com

**Keywords:** synthetic aperture radar (SAR), ground moving target indication (GMTI), radar vision, parameter estimation, road extraction

## Abstract

To detect and estimate ground slowly moving targets in airborne single-channel synthetic aperture radar (SAR), a road-aided ground moving target indication (GMTI) algorithm is proposed in this paper. First, the road area is extracted from a focused SAR image based on radar vision. Second, after stationary clutter suppression in the range-Doppler domain, a moving target is detected and located in the image domain via the watershed method. The target’s position on the road as well as its radial velocity can be determined according to the target’s offset distance and traffic rules. Furthermore, the target’s azimuth velocity is estimated based on the road slope obtained via polynomial fitting. Compared with the traditional algorithms, the proposed method can effectively cope with slowly moving targets partly submerged in a stationary clutter spectrum. In addition, the proposed method can be easily extended to a multi-channel system to further improve the performance of clutter suppression and motion estimation. Finally, the results of numerical experiments are provided to demonstrate the effectiveness of the proposed algorithm.

## 1. Introduction

The detection and motion parameter estimation of ground moving targets in synthetic aperture radar (SAR) have been widely studied in the past decades [1,2,3,4,5,6,7,8,9,10]. For single channel SAR [5], the target can be directly detected and its range velocity can be estimated according to its Doppler centroid, if the Doppler spectrum of a moving target is in the clean region away from that of stationary clutter. However, when a target is moving slowly and its Doppler spectrum is partly submerged by stationary clutter, the estimation performance of Doppler centroid is seriously deteriorated. Meanwhile, the accuracy of locating the moving target in the azimuth direction decreases significantly. In this case, along-track multichannel SAR [6] has been adopted to suppress stationary clutter, but the system complexity of multichannel SAR increases significantly compared to that of single channel SAR. Furthermore, reference [7] proposed a virtual multichannel algorithm based on a real single channel SAR, which has good clutter cancellation results, and is capable of detecting and refocusing slowly moving targets. However, it requires a high pulse repetition frequency (PRF), which will shorten the unambiguous range swath. Also, the azimuth velocity of moving target is another parameter of interest for target motion retrieval, apart from range velocity. The Doppler chirp signal of a moving target induced by azimuth velocity can be presented as a skewed line in the time-frequency plane, for which time-frequency analysis tools, e.g., Wigner-Ville distribution [2] and fractional Fourier transform [9], can be used to estimate chirp rate as well as azimuth velocity. However, these two methods suffer from a heavy computation burden. Normally, ground vehicles move on the roads and their locations are strongly related to the road network. It is straightforward to introduce the idea of the road-aided SAR-GMTI to improve the motion estimation performance, which has been discussed in some previous papers [9,10,11]. In order to estimate the motion parameters, [9] evaluates the intersection points of a moving target with the prior known road axes which are mapped into the range-compressed data domain in advance. Papers [10,11] use along-track interferometry and the displacement of the detected targets from their corresponding road to estimate the across-track velocity. Nevertheless, these existing road-aided approaches need a multichannel SAR system and strongly rely on external sources, e.g., geographic information system (GIS), to obtain road prior knowledge, which increases the SAR system complexity.

To cope with this problem, the concept of radar vision may provide a possible new solution and it was put forward by Haykins in 1990 [12]. The objective of radar vision is to turn radar into a more intelligent remote-sensing device capable of recognizing and exploiting the environment. Most existing research on radar vision has used a radar data and camera visual information fusion approach in fields like obstacle detection and tracking [13], vehicle and unmanned vehicle recognition and classification [14,15]. In this paper, a road-aided method based on radar vision is proposed to make use of the visual information from radar images, which improves the single channel SAR sensing abilities in the ground moving target indication (GMTI). Furthermore, we estimate the azimuth velocity of a moving target from the retrieved range velocity and the extracted road slope via polynomial fitting, which can substantially decrease the computational complexity. In this paper, the signal model is established for an airborne single-channel SAR for a slowly moving target, which moves along an isolated road that is not along the azimuth direction. At first, the road course is extracted from a SAR image by a genetic algorithm [16]. In order to improve the signal-to-clutter ratio (SCR), the stationary clutter is filtered in the range-Doppler (RD) domain. As the slowly moving target signal can be approximately regarded as a 2-D “peak” response in the image domain [6], a watershed algorithm [17,18] can be used to accomplish the target detection and location. With the auxiliary information of the road layout and the azimuth shift direction of the moving target, the target’s position on the road can be determined. Then, the target’s range velocity is acquired in light of the azimuth offset distance. Additionally, the road slope near the target is estimated by polynomial fitting [8]. Finally, the azimuth velocity is obtained with the help of the range velocity and road slope. With the proposed road-aided method based on radar vision, the slowly moving target with more than 40% of Doppler bandwidth out of 3-dB clutter can be readily detected and its 2D velocity can be estimated to correctly locate it in the final SAR images. Compared with the similar algorithm discussed in [7], the proposed algorithm does not need a high PRF and has a lower computation load, but it only can detect and estimate a moving target with a radial velocity larger than the conventional minimum detectable velocity (MDV) which may larger than that described in [7].

The reminder of this paper is arranged as follows: in Section 2, the signal model and imaging algorithm are formulated for a slowly ground moving target in a single channel SAR. In Section 3 a road-aided method based on radar vision is proposed. In Section 4, the results of numerical experiments are provided to verify the proposed method and the performance of proposed algorithm is analyzed. In Section 5, some conclusions are drawn.

## 2. Signal Model of a Slowly Moving Target in SAR

**Figure 1:**
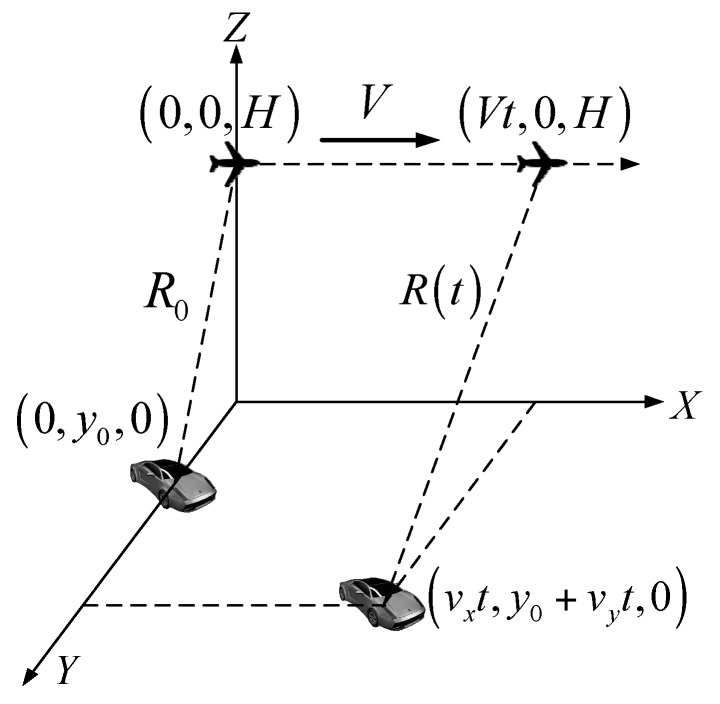
Airborne single-channel SAR system model.

The airborne single-channel SAR system model is presented in Figure 1. The *X*-axis is the azimuth direction, and the *Y*-axis is the range direction. A side-looking SAR platform flies with the constant velocity *V* at the height of *H*. The sampling times of the *X*-axis and *Y*-axis are denoted as *t* and *τ*, respectively. Assume that the coordinate of plane is (0, 0, *H*) and the moving target is located at (0, *y_0_*, 0) when *t* = 0. The target keeps constant azimuth direction velocity *v_x_* and range direction velocity *v_y_* in the synthetic aperture time *T_s_*. The complete signal model of a SAR moving target can be found in [6] with consideration of arbitrary 2D motion speed, and the special case of a slowly moving target is rewritten in this section to provide the signal model for the proposed method in Section 3. The radar sequentially transmits the linear frequency modulation pulses. After demodulation, the base band echo of the moving target is expressed as:

(1) $$s\left(t,\tau \right)=\sigma \text{rect}\left[\frac{\tau -2R\left(t\right)/c}{T_{p}}\right]\text{exp}\left\{j\pi K_{r}{\left[\tau -\frac{2R\left(t\right)}{c}\right]}^{2}\right\}\text{rect}\left(\frac{t}{T_{s}}\right)\text{exp}\left[-j\frac{4\pi R\left(t\right)}{\lambda }\right]$$

where *T_p_* is the time duration, *K_r_* is the modulation rate, *f_c_* is the carrier frequency, rect(⋅) is a rectangular window, *σ* denotes the backscattering coefficient of the target, *λ* = *c*/*f_c_* is the wavelength where *c* is the velocity of light, and *R*(*t*) is the instantaneous range between radar and target given by:

(2) $$R\left(t\right)=\sqrt{{\left(v_{x}t-Vt\right)}^{2}+{\left(y_{0}+v_{y}t\right)}^{2}+H^{2}}\approx R_{0}-\frac{1}{2}\lambda f_{d}t-\frac{1}{4}\lambda K_{a}t^{2}$$

where $R_{0}={R\left(t\right)|}_{t=0}=\sqrt{y_{0}^{2}+H^{2}}$ denotes the initial distance between radar and target when *t* = 0. *K_a_* and *f_d_* are the Doppler chirp rate and Doppler centroid of the moving target given by:

(3) $$K_{a}=-\frac{2\left[{\left(V-v_{x}\right)}^{2}+v_{y}^{2}\right]}{\lambda R_{0}},f_{d}=-\frac{2v_{y}}{\lambda }\frac{y_{0}}{R_{0}}$$

Let $v_{x}=0$ and $v_{y}=0$. The stationary target Doppler chirp rate $K_{a0}$ and Doppler centroid $f_{d0}$ can then be acquired as:

(4) $$K_{a0}=-\frac{2V^{2}}{\lambda R_{0}},f_{d0}=0$$

Considering a slowly moving target with $v_{x},v_{y}<<V$ and $v_{y}<V_{b}$ where $V_{b}=\lambda f_{P}/2$ is the blind speed and *f_P_* is the PRF, *i.e.*, the Type I target in [6], we have $K_{a}\approx K_{a0}$ and generate the signal:

(5) $$s_{rc}\left(t,\tau \right)=\sigma T_{p}\text{sinc}\left\{B\left[\tau -\frac{2R\left(t\right)}{c}\right]\right\}\text{exp}\left[-j\frac{4\pi R\left(t\right)}{\lambda }\right]\text{rect}\left(\frac{t}{T_{s}}\right)$$

where $B=\left|K_{r}\right|T_{p}$ represents the signal bandwidth. Substituting Equation (2) into Equation (5) and after the Fourier transform (FT) of Equation (5) in the azimuth direction, we have:

(6) $$S\left(f_{a},\tau \right)\approx \frac{\sigma T_{p}}{\sqrt{K_{a}}}\text{sinc}\left\{B\left[\tau -\frac{2}{c}\left(R_{0}-\frac{\lambda \left(f_{a}^{2}-f_{d}^{2}\right)}{4K_{a}}\right)\right]\right\}\text{rect}\left(\frac{f_{a}-f_{d}}{B_{a}}\right)\text{exp}\left[-j\pi \frac{{\left(f_{a}-f_{d}\right)}^{2}}{K_{a}}\right]\text{exp}\left(j\varphi \right)$$

where *f_a_* is the azimuth frequency, $B_{a}=\left|K_{a}\right|T_{s}$ is the Doppler bandwidth and $\varphi =-4\pi R_{0}/\lambda -\pi /4$ is a constant phase.

The range migration of both static target and moving target are corrected based on that of static target. The target’s signal after range migration correction in the RD domain is given as:

(7) $$S_{rmc}\left(f_{a},\tau \right)=\frac{\sigma T_{p}}{\sqrt{K_{a}}}\text{sinc}\left[B\left(\tau -\frac{2R_{0}}{c}+\frac{2\Delta r_{m}}{c}\right)\right]\text{rect}\left(\frac{f_{a}-f_{d}}{B_{a}}\right)\text{exp}\left[-j\pi \frac{{\left(f_{a}-f_{d}\right)}^{2}}{K_{a}}\right]\text{exp}\left(j\varphi \right)$$

where $\Delta r_{m}\approx -\lambda f_{d}^{2}/\left(4K_{a}\right)$ is the residual range migration of the moving target.

The matched filtering reference function corresponding to a stationary target is utilized to realize the azimuth focusing of the static scene. Then the signal in the 2-D time domain can be expressed as:

(8) $$s_{image}\left(t,\tau \right)\approx \frac{\sigma T_{p}T_{s}}{\sqrt{K_{a}K_{a0}}}\text{sinc}\left[B\left(\tau -\frac{2R_{0}}{c}+\frac{2\Delta r_{m}}{c}\right)\right]\text{sinc}\left[B_{a}\left(t+\frac{f_{d}}{K_{a}}\right)\right]$$

It can be concluded that targets with small 2-D velocities should have shifted and focused 2-D peak responses in the image domain.

According to Equation (8), the offset distance of a slow moving target in azimuth direction can be estimated as:

(9) $$\Delta X=\left(V-v_{x}\right){\hat{t}}_{m}=-\frac{\left(V-v_{x}\right)v_{y}y_{0}}{{\left(V-v_{x}\right)}^{2}+v_{y}^{2}}\approx -\frac{v_{y}}{V}y_{0}$$

where ${\hat{t}}_{m}=-f_{d}/K_{a}=-v_{y}y_{0}/\left[{\left(V-v_{x}\right)}^{2}+v_{y}^{2}\right]$ denotes the slow time corresponding to the “peak” response. According to Equation (9), it can be found that both *v_x_* and *v_y_* will cause the azimuth shift of the moving target but *v_y_* is a main influencing factor and the azimuth offset is proportional to the range velocity.

## 3. The Proposed GMTI Method Based on Radar Vision

### 3.1. Road Recognition

In light of the concept of radar vision [12], it is important to extract visual information from a focused SAR image to better recognize a scene, e.g., a road where targets move along. Road extraction in a SAR image has been widely studied and lots of effective identification methods have been proposed. Genetic algorithm [16], Hough transform [19] and segment extraction and junction [20] are some typical approaches for SAR road extraction. In this paper, a method proposed in [16] is used, which includes the following six steps: (1) a SAR image is preprocessed by median filtering and binarization. (2) Morphological operators are employed to remove irrelevant parts. (3) Canny operator and edge refining are used for edge detection. (4) Genetic algorithm is adopted for road edge connection. (5) Complete road boundaries are detected by curve fitting. (6) Road surface is filled between the road edge curves.

### 3.2. Stationary Clutter Filtering

In the obtained SAR image, the stationary scene is well focused and acts as strong clutter interference. In other words, it is difficult to directly detect a moving target submerged in the static scene in a SAR image. Therefore, it is necessary to suppress the static clutter in order to increase the SCR before detection. Normally, the spectrum shape of SAR stationary clutter can be regarded as a unimodal and symmetrical type, e.g., Gaussian-shaped, and most of the stationary clutter energy is centered within the 3-dB bandwidth of the spectrum. Therefore, a stationary clutter filter can be designed to suppress clutter by directly discarding the 3-dB bandwidth of the clutter spectrum. Then, two different circumstances occur: in the first case, if the spectrum of a moving target is in the clean region away from that of the stationary clutter, the target energy is completely preserved and the SCR increases after clutter filtering. For the second case, even though part of the spectrum of a moving target is wiped out by the clutter filter, the residual spectrum can be still used to image the moving target with only the azimuth resolution degradation like the sub-look processing [1]. Research shows that as long as more than 40% of target Doppler bandwidth is outside the 3-dB clutter spectrum, the SCR will increase in the image domain after clutter suppression and the moving target can be detected and well estimated, which is studied in detail in Section 4.3.1.

### 3.3. Moving Target Detection

After stationary clutter suppression and the conventional image processing, a SAR image including any moving targets and the residual static scene with a relatively high SCR can be obtained. Then, a watershed algorithm [17,18] is adopted to detect and locate the moving targets in the image. The watershed algorithm treats the SAR image as a 3D landscape under the water. As the water is drained, the local maximal of landscape surfaces will gradually appear and they are assigned as new segments and each segment corresponds to one moving target. The segments will grow when the water is drained until they meet other segments. Finally, the moving targets with Doppler distribution different from the clutter spectrum can be detected and their shifted positions are obtained in the image.

### 3.4. Moving Target Location on the Road

**Figure 2:**
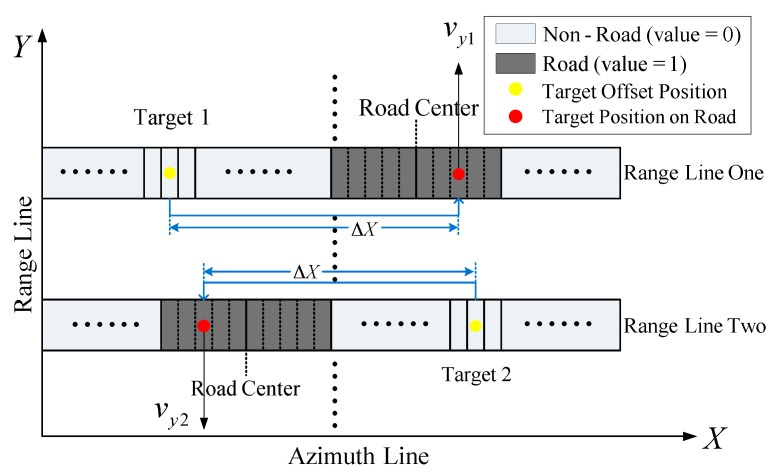
Principle of moving target location on the road.

The road information and the offset position of a moving target shifting from the original real position can be obtained from the joint results of Section 3.1 and Section 3.3. When a moving target moves along the road, the target’s range velocity can accordingly be calculated by Equation (9). The process to locate a target on road is explained in details in Figure 2 by the following steps.

#### 3.4.1. Range Line Extraction

When the moving targets as well as their offset positions have been obtained in the image, all range lines including moving targets can be extracted, like “Range Line One” and “Range Line Two” in Figure 2.

#### 3.4.2. Road Width Calculation

The binary image containing a road is extracted as shown in Figure 2, where the road with value one and the non-road with value zero are used. Clearly, the road width along azimuth can be obtained in pixel in each range line by summing up the values of all the azimuth cells on the range line.

#### 3.4.3. Offset Direction Determination from the Road

As the values of road pixel and non-road pixel are one and zero, respectively, let $\Sigma_{L}$ denote the summation of all azimuth cell values on the left side of a moving target, while $\Sigma_{R}$ is that of the right side of the moving target. If $\Sigma_{L}<\Sigma_{R}$, it means that $\Sigma_{R}$ contains the road part and the offset distance of the moving target from the road ΔX<0 as “Range Line One” in Figure 2. Otherwise, the road is on the left side of the target as “Range Line Two” in Figure 2 when $\Sigma_{L}>\Sigma_{R}$, namely ΔX>0.

#### 3.4.4. Target Range Velocity Direction

According to Equation (9), the direction of the target range velocity can be determined by ΔX. In Figure 2, we have $v_{y1}>0$, *i.e.*, approaching to radar, and $v_{y2}<0$, *i.e.*, leaving away from radar, based on the offset direction obtained in Section 3.4.3.

#### 3.4.5. Estimation of Target Position on the Road

According to a country’s traffic rule, for instance in China, vehicles should move on the right hand side. Hence, the target should move along the middle of a road’s one part for most roads with double lanes. Let’s take Target 1 in Figure 2 for example It is located at the pixel of a quarter to the right edge of the road as *v_y_*_1_ > 0, namely the middle of road’s right part. In the same way, the location of Target 2 on road is determined as the red point in “Range Line Two” as shown in Figure 2. Although in reality the target may not be exactly moving at quarter to the left or right edge of a road, e.g., the road has more than two lanes, the estimation accuracy of range velocity may be reduced but is still acceptable, which will be explained and verified by simulation in Section 4.2.1.

### 3.5. Range Velocity Estimation

With two locations of each target, *i.e.*, the location of detection and the location on the road, the pixel number *N* of azimuth shifting can be obtained. According to Equation (9) and azimuth sampling interval *V*/*f_P_*, the target range velocity is estimated as:

(10) $$v_{y}=-\frac{\Delta X}{\left|\Delta X\right|}\frac{V^{2}}{y_{0}f_{P}}N$$

In addition, according to Section 3.2, the target can be detected and well estimated when more than 40% of the target bandwidth is out of 3-dB clutter. Hence, there is a limit of MDV on radial velocity for a moving target in the proposed algorithm. As it is known, the spectrum bandwidth of 3-dB clutter can be approximated as 2*V*/*D*, where *D* is the antenna aperture. Then, the MDV is given as:

(11) $$v_{y\min}=\frac{0.4V\lambda }{D}$$

### 3.6. Road Slope Estimation

**Figure 3:**
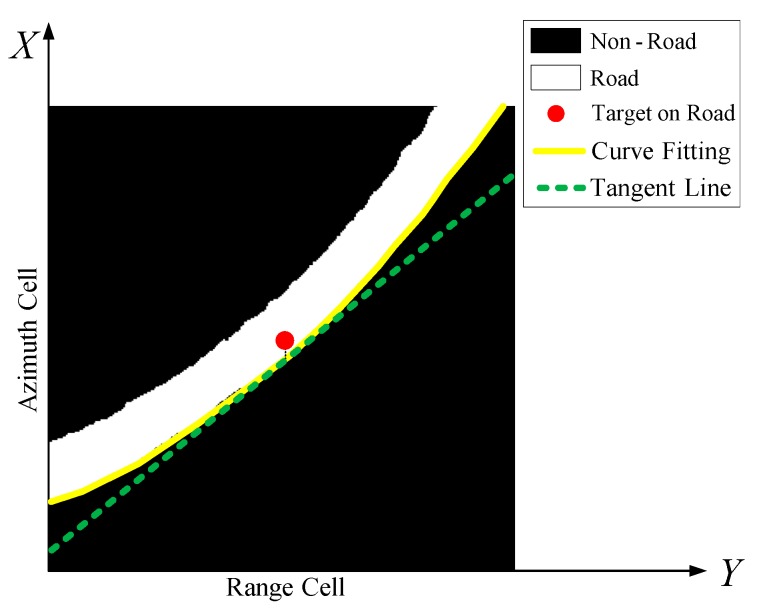
Road slope estimation in the square window.

Based on the obtained range velocity, the road slope can be further used as auxiliary information to estimate the azimuth velocity. The road slope keeps changing with topographical variation. To estimate the adjacent road slope of each moving target, a small square window is employed centering on the moving target position on the road as the binary window in Figure 3. Image processing approaches are utilized in this paper to detect and extract the road edge, and one of the two parallel edges is chosen. In the following step, polynomial fitting [15] is applied to match the road edge curve, which is displayed by the yellow curve in Figure 3. With the linear fitting, the tangent slope of any point on the curve can be easily acquired. Because the window is centered at the target as Figure 3, the tangent slope of the middle point on the curve, *i.e.*, the road slope, can be regarded as the motion slope of the detected target. The green dotted line in Figure 3 is the wanted tangent line and its slope k can be obtained. Furthermore, the slope $k_{Road}$ in 2D imaging plane can be further obtained as:

(12) $$k_{Road}=k\frac{V/f_{P}}{c/2/f_{s}}$$

where *f_s_* denotes the range sampling frequency of the system.

### 3.7. Azimuth Velocity Estimation

Hence, combined with the estimated range velocity *v_y_* and the road slope *k_Road_*, a target’s azimuth velocity *v_x_* can be obtained as:

(13) $$v_{x}=-k_{Road}v_{y}$$

**Figure 4:**
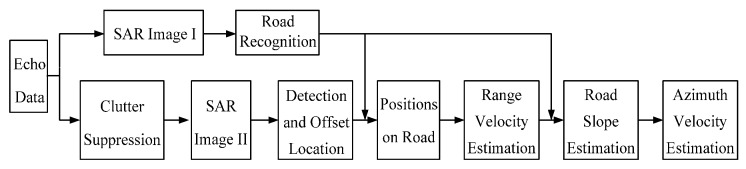
Flow chart of the proposed GMTI method.

The flow chart of the proposed road-aided GMTI method based on radar vision is shown in Figure 4. The method has two aspects. The first is focused on road information extraction from an unfiltered SAR image, and the second is to accomplish the moving target detection and the motion parameter estimation. Obviously, the former provides the necessary aid information for the optimized implementation of the latter.

## 4. Numerical Experiments and Performance Analysis

### 4.1. Numerical Experiments of a Slowly Moving Target

In this section, we investigate the performance of the proposed method based on scene simulations. Radar data is collected by an airborne radar system working in the stripmap mode with the carrier frequency 10 GHz, bandwidth 30 MHz, sampling frequency 37 MHz, platform velocity 200 m/s and pulse repetition frequency 800 Hz. The echo of six moving targets produced by simulation is added to the experimental SAR data of stationary scene and motion parameters set are shown in Table 1.

**Figure 5:**
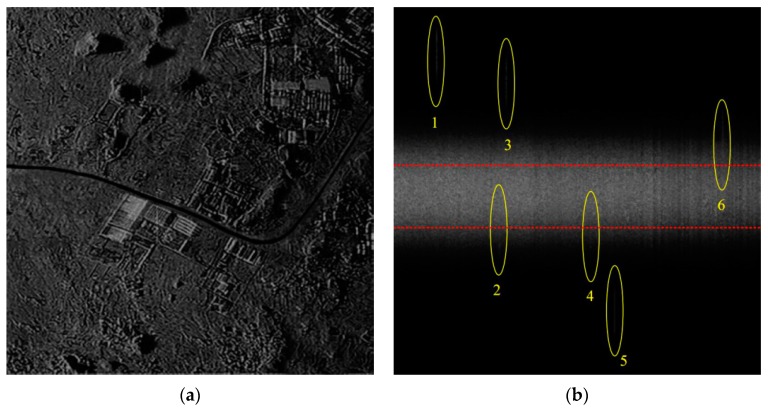
(**a**) The SAR image without 3dB clutter suppression; (**b**) Targets and clutter spectrum in the RD domain.

First, the SAR image without suppressing the stationary clutter is displayed in shown in Figure 5a, where it is very difficult to detect the moving targets in the image because of the interference of the strong background clutter. That is, the SCR needs to be increased to a large extent in order to detect the moving targets. The Doppler spectrum of moving targets and static scene are shown in Figure 5b, in which the region between two dotted red lines is the 3 dB bandwidth of clutter.

**Figure 6:**
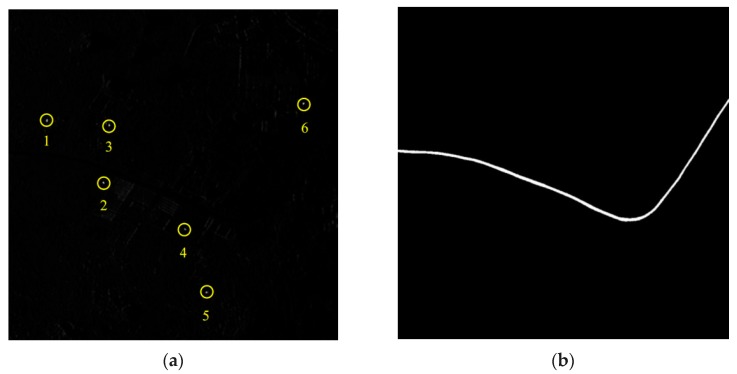
(**a**) The target detection via watershed algorithm; (**b**) The extracted road in the SAR image.

In Figure 6a, the second SAR image after stationary clutter suppression is obtained and the amplitude of the stationary scene greatly decreases. The SCR is increased in the two circumstances mentioned in Section 3.2, which is also verified by the numerical experiments. As a consequence, the moving targets can be detected and located by the proposed watershed algorithm in Section 3.3. The road extraction result is a binary image shown in Figure 6b including white road part and black non-road region. According to road extraction and target azimuth offset direction, the sign of ΔX can be determined and six targets can be relocated on the road accordingly. The azimuth offset distance of each target can be figured out by the two positions in image and the known sampling intervals. According to Equation (10), range velocities are found. Relying on the target positions on road, we use a small square window centering on each target to estimate its adjacent road slope. The value *k_Road_* together with *v_y_* will lead to *v_x_*. All parameters estimated via the radar vision method are shown in Table 1 where it is seen that the detection and parameter estimation of moving targets are acceptable for the proposed method based on radar vision.

### 4.2. Estimation Accuracy Discussion

#### 4.2.1. Estimation Accuracy of Range Velocity

For the proposed method, the range velocity estimation errors arise from three sources, *i.e.*, the formula approximation error, the target offset position error and the target coordinate error on a road. We will analyse them one-by-one.

- Formula approximation error

From the first two terms of Equation (9), the precise azimuth offset has a complicated relationship with target’s 2D motion, *i.e.*, *v_x_* and *v_y_*. Therefore, the approximation used in Equation (9) will inevitably cause an estimation error.

- Offset position error

On the one hand, a slowly moving target can be approximately regarded as a 2-D peak response as Equation (8) and the peak point position can also be determined using Equation (9). However, when the moving target shifts away from its original position, its signal will be superimposed with an unknown scene signal. Therefore, compared with the theoretical peak position, the real peak position may have slight offset, which will also influence the location accuracy. On the other hand, the 3 dB bandwidth of the stationary clutter is suppressed to increase SCR for the proposed method. If the moving target is partially eliminated by the filter, its imaging result will be degraded on the resolution and lead to a slight location error, which will be discussed in detail in Section 4.3.1.

- Location on road error

Each target is located at a quarter to the right or the left edge of road. However, a target may not be exactly at the estimated point position. The road width is limited and may be only a few tens of pixels according to image resolution. If a target moves on the correct side according to traffic rules, the maximum position error will be only a few pixels.

**Figure 7:**
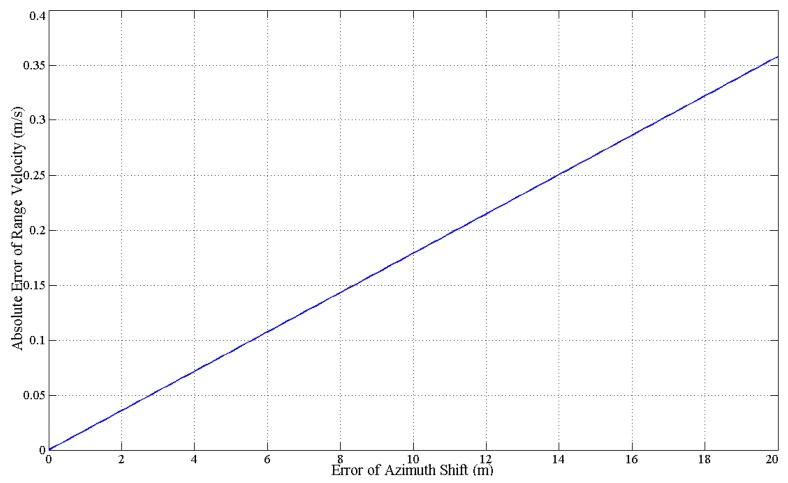
The absolute error of range velocity estimation *versus* the azimuth shift error.

The offset position error and the location on road error can be combined as the error of azimuth shift, and its effect on range velocity estimation is shown in Figure 7. It can be seen from this figure that the absolute error of *v_y_* changes linearly and slightly *versus* the error of ΔX. Even when the error of ΔX rarely reaches 20 m in reality, the absolute error of *v_y_* is only about 0.35 m/s. Hence, under azimuth shift error circumstances, the estimation accuracy of the range velocity may be reduced but is still acceptable.

#### 4.2.2. Azimuth Velocity Estimation Accuracy

Normally, the target moves along the road, and the azimuth velocity estimation error comes from two aspects. The first is the estimation error of the range velocity mentioned above and the second is the estimation error of the road slope near to the moving target. Otherwise, if a moving target suddenly changes its lane during the synthetic aperture time, the motion model becomes too complicated and beyond the scope of this paper.

### 4.3. 2-D Motion Estimation Ability

The main advantage of the proposed method is that it has the ability for 2D motion estimation as follows.

#### 4.3.1. Range Velocity Estimation in a Single-Channel SAR

In the RD domain, if the spectrum of a moving target is in the clean region far away from the stationary clutter, like Targets 1, 3 and 5 in Figure 5b, the range velocity can be estimated effectively by traditional algorithms, such as the energy balance algorithm (EBA) and correlation Doppler estimation (CDE) [21], which is shown in Table 1. However, when the target spectrum is partially submerged by the stationary clutter, e.g., Targets 2, 4 and 6, the accuracies of the range velocity estimation decrease seriously for the EBA and CDE methods as listed in Table 1. On the contrary, even for these targets, the proposed method can still obtain the satisfactory range velocities as shown in Table 1 under some conditions. Furthermore, it is obvious that there are two influential factors that may affect the performance of the proposed algorithm, *i.e.*, the signal-to-clutter ratio (SCR) in the image domain and the Doppler distribution difference between moving target and clutter. In the following part, the above two factors are studied and the signal bandwidth out of clutter ratio (SBOCR) is introduced to describe the extent of the signal Doppler spectrum different from that of clutter spectrum.

**Figure 8:**
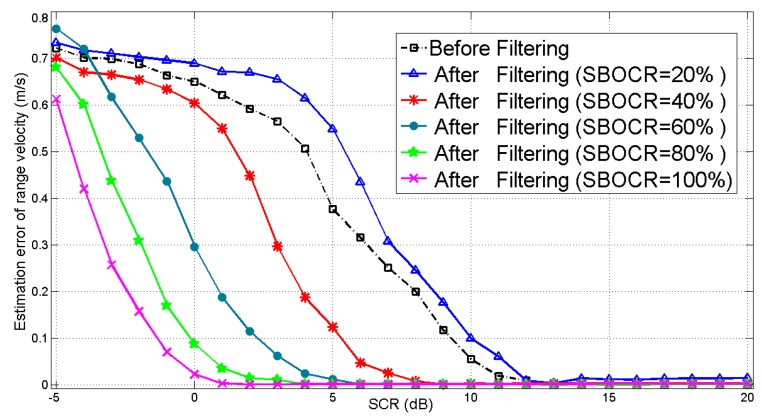
The algorithm performance *versus* SCR and SBOCR.

For the following experiments, Monte Carlo experiments are done 1000 times with different SCRs. First, the range velocity is estimated via the azimuth shift without the 3 dB clutter filtering. Second, the same estimation process is repeated for different SBOCRs after 3 dB clutter suppression. From the experiment results shown in Figure 8, as the SCR increases, the estimation error of the range velocity decreases accordingly. Additionally, the 3 dB clutter filtering is effective because most of the clutter energy is suppressed and the residual signal bandwidth out of the 3 dB clutter range can still image the target well. The higher SBOCR is, the more obvious the estimation performance improvement after clutter filtering is. On the contrary, however, when the SBOCR drops to 20%, which means 80% of moving target signal bandwidth is filtered, the residual energy of moving target signal is extremely low and the performance gets worse after clutter suppression. Therefore, the proposed algorithm works well for moving targets whose signal spectrum is partially submerged in the background clutter with SBOCR from 40% to 100%.

Besides, more targets in different scenes which are omitted for simplicity in this paper have been used to test the effectiveness of the proposed method in addition to Figure 5 and Figure 6.

#### 4.3.2. Azimuth Velocity Estimation

Another advantage of the proposed method is the ability to estimate the azimuth velocity, which is a relatively difficult problem for conventional processing in SAR. In this paper, after the estimation of range velocity from the view of radar vision and with the auxiliary information of road slope, the azimuth velocity can be obtained. It has been shown that the proposed method has a rather low computational complexity and acceptable estimation accuracy.

For the algorithm proposed in this paper, it is assumed that there is only an isolated road in the scene that can be extracted in the image and there is obvious difference on Doppler spectrum between target and clutter, which may not be always satisfied. Our future work will focus on the multiple roads circumstance and try to extend the proposed method to identify some special conditions that define a target moving on a road along the azimuth direction.

## 5. Conclusions

For airborne single channel SAR moving target motion estimation, a novel road-aided algorithm is proposed in this paper. First, the road is extracted as auxiliary visual information from the SAR image based on radar vision. Second, the stationary clutter is directly filtered to increase SCR. Then the watershed method is used to detect and locate moving targets in the image domain. Combined with road information and estimated target positions, the targets are relocated on road using some knowledge about the local traffic regulations. Target range velocity can be obtained according to its corresponding azimuth shifting distance. As for the azimuth velocity, it can be estimated with the help of the road slope near the target via polynomial fitting. Though the estimation accuracy of the traditional algorithms, e.g., EBA and CDE, are better than that of the proposed algorithm for those targets whose spectra are totally distributed away from the strong ground clutter, the proposed method has an advantage over these traditional algorithms when the spectra of moving targets are partially submerged in the clutter. The proposed method can effectively detect and estimate ground moving targets with SBOCR with probabilities ranging from 40% to 100%. Finally, numerical experiment results are provided to demonstrate the effectiveness of the proposed method. Furthermore, it is necessary to emphasize here that the moving target estimation is done via a single SAR image in this paper and different from what is done in the traditional image processing where at least two images are needed for motion estimation.

## Figures and Tables

**Table 1:** Six Moving Targets and Their Motion Estimation.

Target	Motion Parameters		Parameters Estimated					
			The Proposed Method		EBA before Filtering	EBA after Filtering	CDE before Filtering	CDE after Filtering
	*v_y_*(m/s)	*v_x_*(m/s)	*v_y_*(m/s)	*v_x_*(m/s)	*v_y_*(m/s)	*v_y_*(m/s)	*v_y_*(m/s)	*v_y_*(m/s)
1	4.53	0.21	4.5443	0.2036	4.5272	4.5272	4.5317	4.5317
2	−1.25	−0.28	−1.2479	−0.2978	−0.0250	−1.0793	−0.0451	−1.1024
3	3.56	0.79	3.5629	0.7785	3.5588	3.5588	3.5615	3.5615
4	−1.58	−0.47	−1.5758	−0.4524	−0.0051	−1.0886	−0.0107	−1.0035
5	−3.95	−1.10	−3.9573	−1.0793	−3.9536	−3.9536	−3.9472	−3.9472
6	1.85	−1.69	1.8481	−1.7197	0.1260	1.5131	0.1246	1.4938
